# Unveiling the Potential of Sulfur-Containing Gas Signaling Molecules in Acute Lung Injury: A Promising Therapeutic Avenue

**DOI:** 10.3390/cimb46070426

**Published:** 2024-07-07

**Authors:** Xutao Sun, Caiyun Mao, Jiaxin Wang, Siyu Wu, Ying Qu, Ying Xie, Fengqi Sun, Deyou Jiang, Yunjia Song

**Affiliations:** 1 Department of Typhoid, School of Basic Medical Sciences, Heilongjiang University of Chinese Medicine, Harbin 150040, China; sunxutao@hljucm.net; 2 Department of Pharmacology, School of Basic Medical Sciences, Heilongjiang University of Chinese Medicine, Harbin 150040, China; 17737493595@163.com (C.M.); 18741272223@163.com (S.W.); 18646278126@163.com (Y.Q.); 3 Department of Synopsis Golden Chamber, School of Basic Medical Sciences, Heilongjiang University of Chinese Medicine, Harbin 150040, China; wangjiaxin66899@163.com (J.W.); xieying@hljucm.edu.cn (Y.X.); 4 Department of Pathology, School of Basic Medical Sciences, Heilongjiang University of Chinese Medicine, Harbin 150040, China; sunfengqi@hljucm.edu.cn

**Keywords:** hydrogen sulfide, sulfur dioxide, acute lung injury, inflammation, oxidative stress, apoptosis

## Abstract

Acute lung injury (ALI) and its most severe form, acute respiratory distress syndrome (ARDS), are pulmonary conditions that cause significant morbidity and mortality. The common etiologies of these conditions include pneumonia, pulmonary contusion, fat embolism, smoke inhalation, sepsis, shock, and acute pancreatitis. Inflammation, oxidative stress, apoptosis, and autophagy are key pathophysiological mechanisms underlying ALI. Hydrogen sulfide (H_2_S) and sulfur dioxide (SO_2_) are sulfur-containing gas signaling molecules that can mitigate these pathogenic processes by modulating various signaling pathways, such as toll-like receptor 4 (TLR4)/nod-like receptor protein 3 (NLRP3), extracellular signal-regulating protein kinase 1/2 (ERK1/2), mitogen-activated protein kinase (MAPK), phosphatidyl inositol 3 kinase (PI3K)/protein kinase B (Akt)/mammalian target of rapamycin (mTOR), and nuclear factor kappa B (NF-κB), thereby conferring protection against ALI. Given the limited clinical effectiveness of prevailing ALI treatments, investigation of the modulation of sulfur-containing gas signaling molecules (H_2_S and SO_2_) in ALI is imperative. This article presents an overview of the regulatory pathways of sulfur-containing gas signaling molecules in ALI animal models induced by various stimuli, such as lipopolysaccharide, gas inhalation, oleic acid, and ischemia-reperfusion. Furthermore, this study explored the therapeutic prospects of diverse H_2_S and SO_2_ donors for ALI, stemming from diverse etiologies. The aim of the present study was to establish a theoretical framework, in order to promote the new treatment of ALI.

## 1. Introduction

The term acute lung injury (ALI) has been used in a broad sense or in animal models since the introduction of the Berlin definition in 2012. However, it essentially refers to the same pathology as acute respiratory distress syndrome (ARDS), which is characterized by a more severe clinical presentation [1,2]. ALI/ARDS is believed to be primarily caused by hyperpermeability of the alveolar epithelial endothelial barrier, uncontrolled inflammatory reaction, and excessive leukocyte activation [3]. Furthermore, oxidative stress, apoptosis, and autophagy contribute significantly to the pathophysiology of these conditions. ALI etiology encompasses various factors that can directly affect the lungs, such as pneumonia, lung contusion, fat embolism, and smoke inhalation. Additionally, extrapulmonary insults such as sepsis, shock, and acute pancreatitis can also contribute to the development of ALI [4,5]. Emerging research suggests that ALI resulting from direct and indirect lung injury, classified as distinct subtypes of ARDS, exhibits varying responses to therapeutic interventions. This highlights the need for personalized treatment approaches to improve patient outcomes [6,7]. However, existing therapeutic modalities, including protective pulmonary ventilation, prone positioning, and neuromuscular blockade, have proven ineffective in reducing mortality rates [8,9,10].

Sulfur-containing gas signaling molecules such as hydrogen sulfide (H_2_S) and sulfur dioxide (SO_2_) have traditionally been recognized as emissions and atmospheric contaminants characterized by noxious odors. The identification of these gases in mammalian organisms has sparked increased interest in the biological and medical sciences. Current research indicates that H_2_S can influence the NF-E2-related factor 2 (Nrf2) pathway and its associated downstream mechanisms via S-sulfhydrating Keap1. Considering the importance of Nrf2 as a therapeutic target for mitigating the effects of oxidative stress on ALI, these findings could have significant implications for future therapies [11,12]. The anti-inflammatory, antioxidative, and anti-apoptotic properties of H_2_S and SO_2_ have proven to be highly effective in preventing and alleviating ALI and ARDS, promoting lung tissue repair, and reducing mortality rates in patients. Therefore, the aim of the present study was to discuss the regulatory effects of sulfur-containing gas signaling molecules on ALI, in order to provide a theoretical basis for the development of new therapeutic agents and the expansion of therapeutic options for ALI.

## 2. Sources of H_2_S and SO_2_

### 2.1. Endogenous Generation of H_2_S and SO_2_

**Figure 1:**
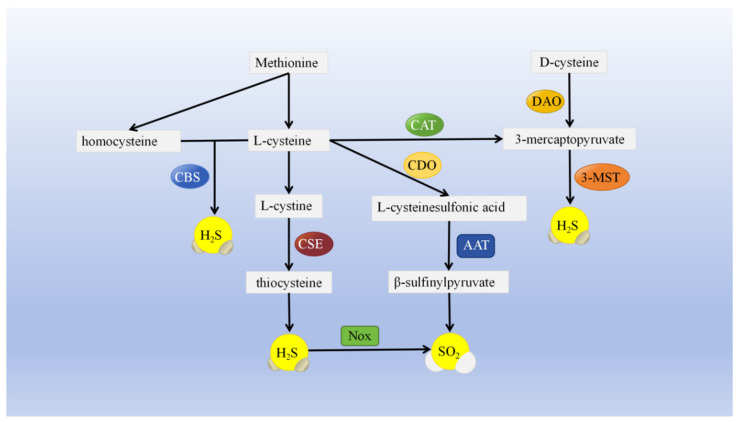
Endogenous production pathways for H_2_S and SO_2_. Sulfur-containing amino acids such as methionine, homocysteine, and cysteine are produced to H_2_S and SO_2_ by enzymes such as CSE, CBS, CAT, DAO, 3-MST, CDO, and AAT. 3-MST: 3-mercaptopyruvate sulfurtransferase; CAT: cysteine aminotransferase; CDO: cysteine dioxygenase; AAT: aspartate aminotransferase; CBS: cystathionine-β-synthase; CSE: cystathionine-γ-lyase; Nox: NADPH oxidase. The synthesis pathways of H_2_S and SO_2_ partially overlap.

The metabolism of sulfur-containing amino acids, including serine, methionine, and cysteine, primarily results in the production of endogenous H_2_S and SO_2_ as the end products. These molecules have low molecular weights, are continuously produced, rapidly diffuse, act independently of membrane receptors, and have extensive effects [13,14]. Cystathionine-β-synthase (CBS), cystathionine-γ-lyase (CSE), and 3-mercaptopyruvate sulfurtransferase (3-MST) are the primary enzymes involved in endogenous H_2_S generation [15] (Figure 1). CBS and CSE directly convert L-cysteine into H_2_S, whereas 3-MST is produced from 3-mercaptopyruvate. L-cysteine and D-cysteine are both sources of 3-mercaptopyruvate, with the former relying on cysteine aminotransferase catalysis and the latter relying on amino acid oxidase [16,17]. Physiologically, methionine can be transformed into cysteine for cellular use via the sulfurization pathway, with CBS and CSE serving as crucial enzymes [18].

The tissue-specific distribution of the three enzymes responsible for synthesizing H_2_S is as follows: CBS is primarily present in the central nervous system, CSE is predominantly located in peripheral organs, and 3-MST is highly active in erythrocytes [19]. CSE exhibits widespread distribution in the heart, liver, spleen, lung, and other peripheral organs. In physiological conditions, endogenous H_2_S plays a regulatory role in cardiac contractility and rhythm, hepatic glucose and fatty acid metabolism, splenic immune cell movement and activation, as well as lung airway tension and gas exchange [19]. The immune response in CSE knockout mice was compromised, leading to decreased phagocytic activity of leukocytes, increased bacterial burden, and reduced survival rates in response to multi-drug resistant Pseudomonas aeruginosa infection [20]. Additionally, ovalbumin-induced airway hyperresponsiveness and inflammation were observed in CSE-deficient mice [21]. Conversely, upregulation of CSE in the cardiovascular system mitigated TNF-α-induced MMP2 hyperactivity and elastolysis, thereby reducing inflammatory infiltration and media degeneration associated with aortic aneurysm [22]. Pulmonary CSE/H_2_S serves as an early regulator in the pathogenesis of ALI. The concentration of CSE in the lung tissue of rats with acute pancreatitis initially increases before decreasing [23]. Pretreatment inhibition of CSE has been shown to mitigate ALI associated with acute pancreatitis. Conversely, prior research indicates that supplementation of H_2_S has a protective effect on lung injury related to pancreatitis [24,25]. Given that CSE is the primary enzyme responsible for H_2_S synthesis in the pancreas, the conflicting outcomes may be attributed to a dynamic regulation of CSE imbalance/balance between the lung and pancreas.

Endogenous SO_2_ is primarily catalyzed by aspartate aminotransferase (AAT), with two isoenzymes, AAT1 and AAT2, found in animals. Cysteine dioxygenase facilitates the conversion of L-cysteine into L-cysteine sulfinate, which is then transformed into β-sulfinylpyruvate by AAT. Eventually, β-sulfinylpyruvate breaks down independently into pyruvate and SO_2_ [26]. In activated neutrophils, NADPH oxidase (Nox) directly oxidizes H_2_S to SO_2_ [27]. Endogenous SO_2_ and its key enzyme, AAT, are present in various rat tissues, including the heart, liver, lung, kidney, and arterial blood vessels. AAT1 and AAT2 are predominantly located in the cytoplasm rather than in the nucleus [28].

### 2.2. H_2_S and SO_2_ Donors

The delivery of exogenous gas signaling molecules is essential for cellular and animal experiments. The most direct method is to inhale gas, although donor compounds are often preferred [29]. Currently, common sources of H_2_S include sulfide salts, GYY4137, diallyl thiosulfinate (allicin), and its oily products derived from hydrolysis, including diallyl sulfide (DAS), diallyl disulfide (DADS), and diallyl trisulfide (DATS). Sodium sulfide (Na_2_S) and sodium hydrosulfide (NaHS) are widely used sulfide salts that rapidly generate H_2_S without byproducts. However, sulfide salts do not accurately simulate the enzymatic generation of endogenous H_2_S because of their rapid release, which leads to uncontrollable and rapid loss [30,31]. In contrast, GYY4137, a recently developed water-soluble H_2_S donor, releases H_2_S gradually and continuously. Allicin and its hydrolysates act as natural H_2_S donors. In addition, there is a class of natural donors namely isothiocyanate, which includes allyl isothiocyanate (A-ITC), erucin (ERU), benzyl isothiocyanate (B-ITC), and 4-hydroxybenzyl isothiocyanate (HBITC), among others [32,33,34]. With advancements in H_2_S studies, some synthetic H_2_S donors have been developed and evaluated in clinical trials. Examples include sildenafil-based H_2_S donors (ACS6), naproxen-based H_2_S donors (ATB-346), and H_2_S-releasing doxorubicin (HS-Dox) [35,36,37]. The number of SO_2_ donors are significantly lower than that of H_2_S donors. In experimental settings, the donors typically consist of a stock solution prepared with SO_2_ gas as well as mixed sulfite of sodium hydrogen sulfite (NaHSO_3_) and sodium sulfite (Na_2_SO_3_) with a molar ratio of 1:3 (NaHSO_3_/Na_2_SO_3_) [38].

## 3. H_2_S for the Prevention and Treatment of ALI

### 3.1. Lipopolysaccharide-Induced ALI

**Figure 2:**
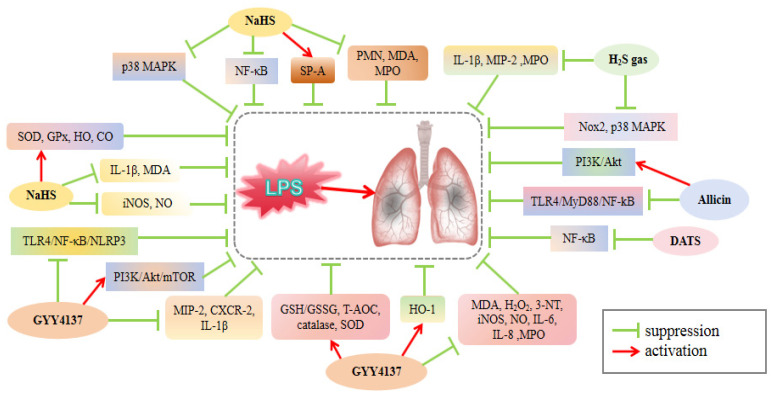
Regulation of H_2_S on LPS-induced ALI. H_2_S donor inclusive of H_2_S gas, allicin, DATS, NaHS and GYY4137 can attenuate LPS-induced ALI by inhibiting the inflammatory pathway NF-κB, activating the autophagy pathway PI3K/Akt/mTOR, and upregulating antioxidant enzymes such as SOD and GPx. 3-NT: 3-nitrotyrosine; Akt: protein kinase B; CXCR2: C-X-C motif-chemokine receptor 2; GPx: glutathione peroxidase; IL-1β: interleukin 1β; iNOS: inducible nitric oxide synthase; MAPK: mitogen-activated protein kinase; MDA: malondialdehyde; MIP-2: macrophage inflammatory protein-2; MPO: myeloperoxidase; mTOR: mammalian target of rapamycin; MYD88: Myeloid Differentiation Primary Response 88; NF-κB: nuclear factor kappa B; NLRP3: nod-like receptor protein 3; PI3K: phosphatidyl inositol 3 kinase; PMN: polymophonuclear neutrophil; SOD: superoxide dismutase; T-AOC: total antioxidant capacity; TLR4: toll-like receptor 4; HO-1: heme oxygenase-1; Nox: NADPH oxidase; DATS: diallyl trisulfide; SP-A: surfactant protein A.

Lipopolysaccharide (LPS), a glycolipid present in the cell wall of Gram-negative bacteria, can trigger severe inflammatory responses in the airways and lungs [39]. Reducing pulmonary surfactants is a crucial physiological and pathological process of LPS-induced ALI [40]. Because of the clinical correlation between these manifestations and ALI/ARDS, LPS-induced ALI animal models have been extensively used to investigate human ALI/ARDS. Mice and rats are commonly used experimental animals in these studies. Studies have suggested that downregulation of the H_2_S/CSE system may be associated with the pathogenesis of LPS-induced ALI (Figure 2 and Table 1). Zhou et al. [41,42] observed reduced H_2_S concentrations in the plasma and decreased CSE activity in lung tissue of LPS-treated rats. LPS also increased inducible nitric oxide synthase (iNOS) enzyme activity and stimulated nitric oxide (NO) production in the plasma. NaHS pretreatment alleviated LPS-induced changes, whereas DL-propargylglycine (PAG, a CSE inhibitor) pretreatment aggravated lung injury. Several studies have confirmed the ability of H_2_S to improve LPS-induced ALI in various ways, highlighting its significant therapeutic potential. Wang et al. [43] found that CSE activity, H_2_S content, and pulmonary surfactant protein A (SP-A) mRNA expression were significantly increased in rats when they were administered different levels of NaHS. However, addition of PAG resulted in a decrease in SP-A, SP-B, and SP-C, suggesting that exogenous H_2_S could potentially improve ALI by regulating the composition and release of pulmonary surfactants.

The anti-inflammatory effects of H_2_S play a pivotal role in preventing the development of ALI. Direct inhalation of 80 ppm H_2_S in LPS-treated mice resulted in reduced ALI scores, inhibited alveolar wall thickening and cell infiltration, and reduced the levels of the pro-inflammatory cytokines interleukin 1β (IL-1β), macrophage inflammatory protein-2 (MIP-2), and serum myeloperoxidase (MPO) glycoprotein, a marker of neutrophil activity. H_2_S exerts its protective effects by inhibiting neutrophil migration and pro-inflammatory cytokine release [53]. Further investigations have revealed that inhalation of additional doses of H_2_S can not only decrease the histopathological damage, neutrophil recruitment, and IL-1β release, but also reduce the protein levels of CBS, HSP70, phosphorylated p38 mitogen-activated protein kinase (p-p38 MAPK), and Nox2 in lung tissues. Inhalation can also inhibit reactive oxygen species (ROS) production [48]. These results indicate that H_2_S can limit ROS production by inhibiting the Nox2 and p38 MAPK signaling pathways, leading to anti-inflammatory and antioxidative effects that prevent LPS-induced ALI. Similarly, the preventative use of sustained-release H_2_S compound GYY4137 decreased the mRNA expression of MIP-2 and its receptor CXCR-2, two mediators of neutrophil migration, in mouse lung tissues. This reduction in expression led to a decrease in the build-up of MIP-2 and IL-1β in the alveolar space. In vitro results have also shown that GYY4137 exerts a direct inhibitory effect on Hoxb8 neutrophils, preventing their movement, cytokine excretion, and ROS generation [47]. Furthermore, GYY4137 decreased the levels of IL-6, IL-8, and MPO in the mouse lungs, restored the inhibitory effect of LPS on the anti-inflammatory cytokine IL-10, and mitigated LPS-induced lung inflammation [49]. These findings further suggest that H_2_S can inhibit neutrophil stimulation, movement, cytokine secretion, and oxidative stress during LPS exposure, ultimately leading to a reduction in lung tissue inflammation and injury.

Heme oxygenase-1 (HO-1) and its byproduct carbon monoxide (CO) have shown potential therapeutic benefits in animal models of ALI by cutting proinflammatory pathways [89]. In a mouse model of lung injury, GYY4137 upregulated HO-1 and suppressed iNOS and COX-2 expression. These effects were reversed when the HO-1 inhibitor SnPP was used [59]. These findings indicate that H_2_S can protect lung tissue from LPS-induced injury by reducing the inflammatory response through the upregulation of HO-1. H_2_S can also reduce the expression of proinflammatory cytokines by downregulating nuclear factor kappa B (NF-κB), resulting in the inhibition of inflammatory reactions. For example, DATS pretreatment prevents ALI by inhibiting NF-κB activation and reducing the release of tumor necrosis factor-α (TNF-α) in the serum and homogenized lung fluid of mice [55]. Allicin treatment in neonatal rats with ALI suppressed the levels of TNF-α, IL-1β, IL-6, IFN-γ, COX-2, and NF-κB in lung tissue [50]. Allicin was administered to the rats at varying concentrations (25, 50, and 100 μg/mL). The results demonstrated significant improvements in lung injury and inflammatory cell infiltration across all dosage groups as well as a notable reduction in apoptotic cells in the lung tissue. Immunohistochemical analysis revealed a significant decrease in the activity of toll-like receptor 4 (TLR4), myeloid differentiation primary response 88 (MyD88), NF-κB, caspase-3, and caspase-9 in lung tissue following allicin treatment. These findings suggest that allicin may mitigate ALI by modulating the TLR4/MyD88/NF-κB pathway in a dose-dependent manner [52].

Mitochondrial dysfunction significantly influences cell damage. Recent research has indicated that the regulation of mitochondrial quality is crucial for attenuating LPS-induced ALI [90]. The rats were injected with LPS via the sublingual vein and exposed to varying doses of NaSH (0.78, 1.56, and 3.12 mg/kg). Compared with the LPS injury group, a significant decrease in serum IL-1β level, mitochondrial malondialdehyde (MDA) concentration, and degree of mitochondrial swelling were observed. Additionally, there was a considerable increase in the activities of ATPase, superoxide dismutase (SOD), and glutathione peroxidase (GPx) [44,45]. These changes were dose-dependent, suggesting that H_2_S can reduce mitochondrial lipid peroxidation, protect cell structure and function, and exhibit effects that are proportional to the dose. The antioxidant properties of H_2_S also directly protect the lung cells from LPS-induced oxidative stress. Zhang et al. [49] found that GYY4137 treatment effectively reversed LPS-induced oxidative and nitration stress in mouse lungs, which was demonstrated by a reduction in MDA, H_2_O_2_, and 3-NT levels, as well as inhibition of iNOS expression and NO production. Additionally, GYY4137 treatment increased the levels of antioxidant biomarkers such as the glutathione (GSH)/GSSG ratio, T-AOC, catalase, and SOD. In rats, allicin treatment reduced the level of MDA in the serum, increased the activities of SOD, GSH, and GPx, and alleviated oxidative stress [50]. These results indicate that H_2_S exerts a beneficial effect on the lung tissue by combating oxidative stress and reducing inflammation.

Studies have demonstrated that preventing and delaying polymorphonuclear neutrophil (PMN) apoptosis can result in the prolonged release of their products, causing tissue damage and contributing to the persistence of ALI [91]. Therefore, enhancing PMN apoptosis can reduce lung inflammation and injury. Both in vitro and in vivo experiments on LPS-induced ALI in rats suggest that NaHS can reduce pulmonary microvascular permeability and ameliorate ALI by inducing PMN apoptosis, which helps minimize PMN accumulation in the lung tissue [57]. Fan et al. [51] reported that administering NaHS via pre-intraperitoneal injection to rats resulted in a reduction in the number of PMN cells in the alveolar septum, as well as a decrease in MPO activity, MDA content, and protein expression of intercellular adhesion molecule-1 (ICAM-1) and p-p38 MAPK in lung tissue. In contrast, pre-injection of PAG increased the number of PMN in the alveolar septum. In addition, a strong association was observed between p-p38 MAPK and ICAM-1 levels in lung tissues. H_2_S inhibits the p38 MAPK pathway, which reduces ICAM-1 protein expression on endothelial cell surfaces, promotes PMN apoptosis, and reduces PMN aggregation in the lungs. Rats treated with NaHS via gavage had lower ALI severity, PMN cell counts, and ICAM-1 expression. The difference is that, consistent with ICAM-1 downregulation, a reduction in NF-κB expression was detected. Therefore, the mechanism by which NaHS reduces PMN accumulation in the lung may also be associated with inhibition of the NF-κB pathway, resulting in the reduction of ICAM-1 expression [58].

Autophagy is a crucial mechanism that transports defective proteins and organelles to the lysosomes for degradation and reuse. It significantly contributes to the pathogenesis of ALI and various pulmonary diseases [92,93]. The phosphatidyl inositol 3 kinase (PI3K)/protein kinase B (Akt) pathway regulates mammalian target of rapamycin (mTOR), the primary inhibitor of autophagy [94]. Research has indicated that administration of GYY4137 can effectively alleviate the inhibitory effects of LPS on the PI3K/Akt/mTOR signaling pathway in mice. It also decreases Beclin1 levels and the conversion of LC3I to LC3II [54,78]. Simultaneously, H_2_S and 3-methyladenine, an autophagy inhibitor, effectively reduced LPS-induced alterations in lung tissue, protein levels in bronchoalveolar lavage fluid (BALF), and TNF-α and IL-1β levels [54]. These findings suggest that H_2_S may alleviate ALI by inhibiting autophagy through the PI3K/Akt/mTOR pathway. In addition, H_2_S attenuates LPS-induced ALI by reducing apoptosis via the PI3K/Akt pathway. Wang et al. [50] found that in the ALI neonatal rat model, allicin treatment increased the expression of PI3K, phosphorylated Akt (p-Akt) and Bcl-2 proteins, and reduced caspase-3/-9 activity and cell apoptosis. Activation of HO-1 via the PI3K/Akt pathway has been identified as a key element in the management of sepsis-induced ALI [90]. H_2_S donors can activate PI3K/Akt [50,54,78] and increase HO-1 expression [59,89], indicating their potential as treatments for sepsis-induced ALI. Consequently, H_2_S may have therapeutic potential in sepsis-induced ALI via the PI3K/Akt/HO-1 pathway, although further experimental confirmation is necessary. H_2_S can modulate Nrf2 and its downstream *HO-1* gene expression by S-sulfhydrating Keap1 [11], and this potential effect should be controlled in experiments, with Nrf2^−/−^ mice serving as a suitable reference.

Methionine restriction (MR) has been demonstrated to significantly reduce organ damage, including LPS-induced pulmonary edema, hemorrhage, atelectasis, and alveolar epithelial cell damage. Additionally, it can block the TLR4/NF-κB/nod-like receptor protein 3 (NLRP3) pathway, thereby reducing IL-1β, IL-6, and TNF-α release and preventing immune cell infiltration [46]. Endogenous H_2_S is crucial in this process because the decrease in methionine following MR stimulates cysteine production, leading to higher levels of CSE and CBS enzymes [95]. Consistent findings demonstrate that inhibition of CSE protein expression reverses the protective effect of MR on ALI. Additionally, GYY4137 treatment in CSE^−/−^ mice mimicked the protective effect of MR, suggesting that CSE and H_2_S play critical roles in decreasing LPS-induced lung injury [46]. Similarly, cholecystokinin 8 (CCK-8), a significant endogenous functional component of the CCK family, mitigated lung tissue injury in rats with ALI. This treatment lowered the quantity and protein levels of PMN in BALF, as well as MDA levels and MPO activity in lung tissues. Furthermore, CCK-8 treatment increased CSE mRNA expression and protein catalytic function, leading to an increase in H_2_S levels in lung tissue. This suggests that CCK-8 also relies on the action of H_2_S to alleviate ALI [56].

In summary, H_2_S demonstrates beneficial effects in animal models of LPS-induced ALI. H_2_S functions through various mechanisms including anti-inflammatory actions via suppression of the TLR4/MyD88/NF-κB pathway, antioxidant effects by inhibition of the p38 MAPK pathway, modulation of autophagy through activation of the PI3K/Akt/mTOR pathway, and prevention of alveolar cell apoptosis via activation of the PI3K/Akt pathway. Additionally, H_2_S reduces ICAM-1 expression by inhibiting the p38 MAPK and NF-κB pathways, leading to the promotion of PMN apoptosis. Furthermore, the LPS-induced ALI model has demonstrated the ability to replicate a range of clinicopathological characteristics associated with ALI/ARDS and remains the most extensively researched model in this field. Given that LPS is derived from the cell wall of Gram-negative bacteria, it is commonly utilized to mimic ARDS induced by sepsis. Despite the substantial volume of literature on LPS-induced lung injury, experimental studies continue to predominantly involve small rodent models. Therefore, there is a need to extend this research to larger animal models, such as sheep and pigs.

### 3.2. Gas Inhalation-Induced ALI

**Figure 3:**
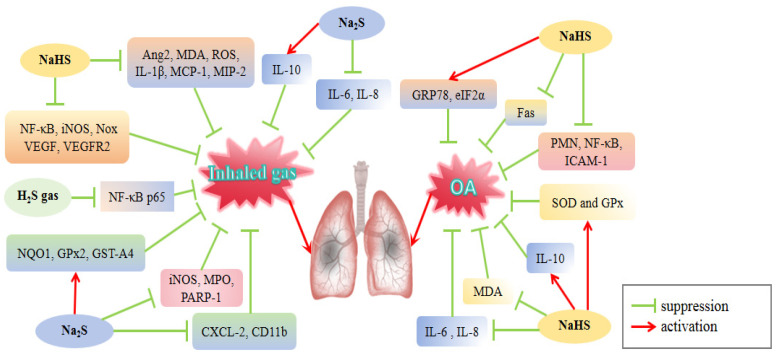
Regulation of H_2_S on gas inhalation/OA-induced ALI. Ang2: angiopoietin-2; NQO1: NAD(P)H: quinone oxidoreductase; PARP-1: poly (ADP-ribose) polymerase-1; ROS: reactive oxygen species; VEGFR2: vascular endothelial growth factor receptor 2; GST-A4: glutathione-s-transferase a4; MCP-1: macrophage chemoattractant protein-1; ICAM-1: intercellular adhesion molecule-1; GRP 78: glucose-regulated protein 78; eIF2α: eukaryotic translation initiation factor-2. H_2_S donors such as H_2_S gas, NaHS, and Na_2_S improved both ALI models induced by inhaled gas and OA by down-regulating the pro-inflammatory factors IL-6 and IL-8 and up-regulating the antioxidant enzymes SOD and GPx.

Inhalation of toxic and damaging gases and chemicals present in smoke contributes significantly to lung injury. Exposure of the lung parenchyma to these detrimental components can cause inflammatory responses and increase the production of MDA, NO, iNOS, and γ-GCS. However, antioxidants have been shown to reduce lung injury [96,97]. H_2_S has been hypothesized to protect against smoke-induced ALI by activating the anti-inflammatory and antioxidant pathways (Figure 3). Specifically, Na_2_S treatment decreased IL-1β levels and increased IL-10 levels in the lung tissue of an ALI mouse model induced by burn combined with cotton smoke inhalation [61]. The histological condition of the lungs improved and the mortality rate of the mice was significantly reduced. Alternatively, Na_2_S administration attenuated protein oxidation after injury. On this basis, they established an ALI model in sheep and found that Na_2_S treatment reduced protein oxidation, as well iNOS, MPO, and peroxynitrite levels, as well as PARP-1 activity. Consistently, sheep survival rates increased significantly, pulmonary gas exchange improved, and burn and smoke-induced increases in inspiratory pressure and fluid accumulation were suppressed during the 96-h experimental period after Na_2_S administration [62]. A study on rats exposed to cotton smoke inhalation indicated that inhalation of 80 ppm H_2_S reduced the levels of MDA, NO, and NF-κB p65 in the lung tissue and inhibited iNOS mRNA and protein expression [60]. H_2_S may reduce iNOS transcription and expression and NO generation by blocking NF-κB p65 activation, which could potentially alleviate cotton smoke inhalation-induced lung damage.

Oxygen therapy is the most common treatment for hypoxemia; however, prolonged exposure to elevated oxygen levels can cause hyperoxic ALI (HALI) [98,99]. Hyperoxia causes lung injury in mice, leading to elevated levels of MDA, ROS, and peroxynitrite as well as upregulation of IL-1β, MCP-1, MIP-2, and angiopoietin-2 (Ang2). NaHS treatment in mice inhibited these indicators [64,65]. Furthermore, Li et al. [64] found that the administration of NaHS to HALI mice led to a reduction in NF-κB activity and iNOS expression, as well as an enhancement in Nrf2 nuclear translocation, resulting in elevated HO-1 activity and reduced Nox activity. These findings suggest that exogenous H_2_S inhibits oxidative stress and inflammation, and alleviates hyperoxia-induced ALI. H_2_S treatment also decreased lung permeability and inhibited the release of VEGF and VEGFR2 in the oxygen-exposed mice. Additionally, NaHS reduced the number of PMN cells and Nox protein content in BALF, decreased apoptosis, and extended the lifespan of hyperoxia-exposed mice [64]. H_2_S preconditioning had a protective effect against hyperoxia-induced lung damage. In rats with hyperoxia-induced ALI, Liu et al. [63] found that pretreatment with NaHS improved lung tissue morphology and reduced IL-13 levels in BALF and TUNEL-positive cells.

Ventilators are used for life support; however, their inappropriate usage may cause lung injury [100]. Although irritation and cytotoxicity of H_2_S gas to the airway mucosa have long been reported [101,102], exposure to 80 ppm of H_2_S during mechanical ventilation at 12 mL/kg tidal volume in mice protects against ventilator-induced lung injury (VILI) [103]. To further understand the function of H_2_S in VILI, Francis et al. [66] established a mouse model of VILI and administered H_2_S during a high tidal volume ventilation of 40 mL/kg. Inhaling one or five ppm H_2_S had no effect on lung injury; however, inhalation of 60 ppm H_2_S accelerated the onset of VILI and increased the expression of CXCL-2, CD11b (integrin adhesion molecule), and L-selectin. Conversely, prior administration of intravenous Na_2_S reduced CXCL-2 and CD11b levels under conditions of high tidal volume ventilation, decreased IL-6 concentrations in BALF, and mitigated pulmonary edema. Additionally, Na_2_S increased the levels of Nrf2-dependent antioxidant genes (*NQO1*, *GPx2*, and *GST-A4*) and protected the lung tissues from oxidative stress-induced glutathione depletion (Figure 3). These findings suggest that instead of direct lung exposure to H_2_S gas, intravascular infusion of Na_2_S may be a more effective therapeutic strategy to prevent VILI.

In conclusion, H_2_S demonstrates a protective role in gas-induced ALI primarily through its anti-inflammatory and antioxidant properties. H_2_S suppresses lung inflammation by inhibiting NF-κB p65 activation and mitigates oxidative stress damage by upregulating antioxidant enzyme expression downstream of Nrf2. In clinical settings, patients with ALI/ARDS often receive oxygen therapy via ventilators or other passive systems as adjunctive treatment, which may inadvertently contribute to lung injury due to the limitations of this therapeutic approach. The aforementioned results indicate that H_2_S preconditioning is efficacious in mitigating the detrimental effects of this treatment, with intravenous sulfide salt infusion demonstrating greater effectiveness compared to direct administration of H_2_S gas during ventilation.

### 3.3. Oleic Acid-Induced ALI

Oleic acid (OA)-treated animal models are commonly used to study ALI because of OA’s ability to induce the production of key inflammatory mediators associated with clinical ARDS [104]. Following OA treatment, rats showed a reduction in H_2_S content in the plasma and lungs, with further decreases observed as ALI progressed [67,69]. In contrast, NaHS administration led to an increase in H_2_S content in the lung tissue [70,71]. OA treatment significantly increased IL-6 and IL-8 concentrations and decreased IL-10 levels in plasma and lung tissues; however, intraperitoneal injection of NaHS reversed the above results and reduced PMN infiltration and the severity of ALI [69] (Table 1), suggesting a potential association between OA-induced ALI development and decreased endogenous H_2_S production.

In another study using an OA-induced ALI rat model, injection of NaHS into the peritoneum decreased the percentage of PMN cells in BALF and reduced the nuclear expression of NF-κB and membrane level of ICAM-1 in alveolar epithelial cells. This suggests that H_2_S may have an anti-inflammatory effect on OA-induced ALI by suppressing NF-κB activation [70] (Figure 3). Wang et al. [103] discovered that injecting NaHS into the peritoneum of rats led to higher levels of H_2_S, SOD, and GSH in lung tissue, while decreasing the levels of MDA in both plasma and lung tissue. These findings imply that H_2_S can reduce OA-induced ALI by inhibiting oxidative stress. Targeting apoptosis is crucial for ALI management. Liu et al. [67] found that pretreatment with an intraperitoneal injection of NaHS reduced the percentage of PMN cells in BALF, alveolar epithelial cell apoptosis, and Fas protein expression in ALI rats, supporting the hypothesis that H_2_S protect rats from lung injury by inhibiting apoptosis. Moreover, NaHS pretreatment increased the levels of GRP78 and eIF2α in alveolar epithelial cells, particularly at 4 and 6 h, indicating that the beneficial effect of H_2_S on ALI could be linked to the elevation of endoplasmic reticulum stress proteins [68].

The aforementioned results suggest that H_2_S may mitigate the inflammatory response triggered by OA through the inhibition of the NF-κB pathway, thereby demonstrating anti-inflammatory properties. Additionally, the rat model of OA-induced ALI was utilized to mimic the clinical manifestation of ARDS induced by lipid embolism, and the beneficial impact of NaHS pretreatment on rats underscores the therapeutic promise of H_2_S. Future studies should aim to replicate these findings in larger animal models for further investigation.

### 3.4. Other ALI Models

**Figure 4:**
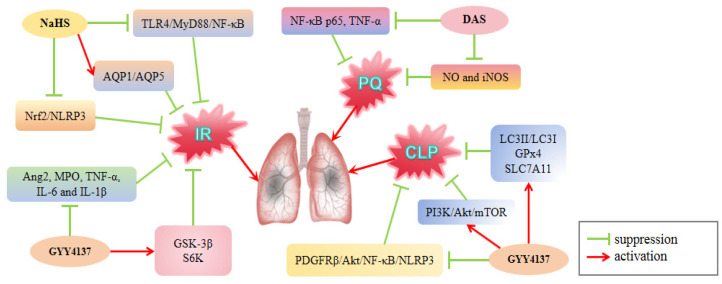
Regulation of H_2_S on I/R, PQ and CLP-induced ALI. Nrf2: NF-E2-related factor 2; SLC7A11: solute carrier family 7 member 11; PQ: paraquat; CLP: cecal ligation and puncture; IR: ischemia reperfusion; GSK-3β: glycogen synthase kinase 3β; S6K: ribosomal protein s6 kinase; PDGFRβ: platelet-derived growth factor Rβ-chain; AQP1: aquaporin 1. H_2_S donors such as DAS, NaHS and GYY4137 improved three ALI models, which are induced by PQ, IR and CLP, respectively, by inhibiting the inflammatory pathway NF-κB and activating the autophagy pathway PI3K/Akt/mTOR.

I/R injury is a pathological condition in which surgical intervention induces a systemic inflammatory response that might cause damage to distal vulnerable organs, such as the lungs [105]. Qi et al. [74] found that treating rats with NaHS after lower limb ischemia-reperfusion (LIR) can increase the levels of aquaporin 1 (AQP1)/AQP5, suppress the TLR4/MyD88/NF-κB pathway, and alleviate lung damage. However, PAG administration had the opposite effect, aggravating lung injury. These findings indicate that H_2_S can reduce LIR-induced inflammatory responses by modulating the TLR4/MyD88/NF-κB and AQP1/AQP5 pathways, leading to protection against ALI (Figure 4). Infrarenal aortic cross-clamping (IAC) is a type of infrarenal blood vascular surgery that can cause I/R injury in the lower extremities and distant organs, such as the lungs [106] (Table 1). Tang et al. [73] found that administering GYY4137 before IAC exposure significantly reduced the severity of ALI, significantly attenuated IAC-induced ALI, lowered MPO activity, and decreased levels of inflammatory cytokines TNF-α, IL-6, and IL-1β in lung tissues. In addition, GYY4137 attenuated Ang2 secretion, promoted p-Akt activity, and activated the downstream factors, GSK-3β and S6K. In contrast, PAG treatment had the opposite effect to that of GYY4137, worsening the severity of lung injury. This suggests that H_2_S protects lung function after IAC by inhibiting Ang2 release and inflammatory response. Furthermore, Zhao et al. [72] found in the ALI mouse model induced by renal ischemia-reperfusion (RIR) that the administration of NaHS improved lung injury and significantly increased the expression of HO-1, NQO1 and Trx, the downstream signaling molecules of Nrf2. In addition, it decreased the protein and mRNA levels of NLRP3, caspase-1, and IL-1β. However, these effects of NaHS were abolished in the Nrf2^−/−^ mice. This demonstrated that NaHS ameliorated RIR-induced ALI by promoting Nrf2 activation, which inhibited the NLRP3 pathway. In summary, in a model of I/R-induced ALI, H_2_S enhances the clearance of edema fluid by up-regulating aquaporin water channel proteins AQP1/AQP5, mitigates lung inflammation by suppressing the TLR4/MyD88/NF-κB p65 signaling pathway, and attenuates oxidative stress by promoting the expression of downstream antioxidant enzymes regulated by Nrf2. These findings indicate that H_2_S confers protection against I/R-induced ALI.

Paraquat (PQ) is a widely used herbicide that accumulates in the lungs after entering the human body and does not decrease with plasma clearance. Every year, numerous people die because of active or accidental ingestion [107]. The molecular mechanism of PQ toxicity involves redox cycling, in which NO is also involved [108]. Cao et al. [75] found that in the ALI model of rats caused by PQ poisoning, the levels of iNOS mRNA and NO secreted by alveolar macrophages in the PQ group were significantly higher than in the control group. Additionally, the pathological injury score of the rat lung tissue increased significantly. Following DAS treatment, the data revealed a significant reduction, indicating that DAS may protect against lung damage in rats exposed to PQ (Figure 4). Furthermore, intraperitoneal administration of DAS in rats decreased inflammatory cell infiltration and suppressed NF-κB p65 activation and TNF-α mRNA in the lung tissue, providing additional evidence that H_2_S may mitigate the severity of lung damage caused by PQ exposure [109].

Animal models of lung damage caused by cecal ligation and puncture (CLP) are frequently used to investigate sepsis-induced ALI. GYY4137 treatment has been shown to minimize neutrophil infiltration, ameliorate septic lung tissue pathology, and reduce lung tissue injury in a CLP-induced ALI mouse model [77,78]. Additionally, the efficacy of 50 μg/g was greater than that of 25 μg/g, indicating that the protective effect of GYY4137 was dose dependent. GYY4137 also decreased the expression of p-PDGFRβ, p-NF-κB, ASC, NLRP3, caspase-1, and p-Akt in the lung tissues of septic mice. GYY4137 may alleviate CLP-induced ALI in mice by inhibiting the PDGFRβ/Akt/NF-κB/NLRP3 pathway [27]. In addition, GYY4137 attenuates sepsis-induced ALI ferroptosis by increasing GPx4 and SLC7A11 levels in lung tissues following CLP [78].

ACS15, a diclofenac derivative that releases H_2_S, demonstrated significant efficacy in mitigating lung inflammation in a mouse model of cerulein-induced acute pancreatitis and related lung damage. Prophylactic and therapeutic administration of ACS15 effectively inhibited alveolar thickening and neutrophil infiltration, whereas diclofenac treatment alone provided limited protection. These findings suggest that ACS15’s efficacy in treating pancreatitis-induced lung damage is attributable to its H_2_S component [27]. When the mouse model was treated directly with different doses of NaHS (5, 10, and 15 mg/kg), 10 mg/kg NaHS treatment significantly alleviated the inflammatory response of the lung and decreased chemokines, such as CCL2 and CXCL1, as well as adhesion molecules, such as P-selectin, ICAM-1, and VCAM-1. Compared with 10 mg/kg, NaHS at 5 mg/kg did not demonstrate a significant increase in effectiveness, and a higher dose of 15 mg/kg provided no additional protective benefits. Therefore, 10 mg/kg NaHS may be a suitable therapeutic dose, and its anti-inflammatory effect is related to the reduction in chemokines and adhesion molecules in the lungs [28].

In conclusion, H_2_S demonstrates potential in reducing PQ-induced ALI through the inhibition of NF-κB p65 activation, as well as in reducing CLP-induced ALI by inhibiting the PDGFRβ/Akt/NF-κB/NLRP3 pathway. PQ, CLP or pancreatitis-induced ALI model is more consistent with clinical ALI. The protective effects of various H_2_S donors such as DAS, GYY4137, and NaHS on ALI models induced by PQ and other etiologies suggest that the clinical application of H_2_S may serve as a promising therapeutic approach for ARDS. However, the translation of findings from animal studies to human clinical settings poses a significant challenge.

## 4. SO_2_ for the Prevention and Treatment of ALI

Early studies have shown that prior administration of glucocorticoids (GC) can prevent or attenuate LPS-induced lung damage [110]. Datzmann et al. [80] used an ALI model induced by atherosclerotic hemorrhagic shock in swine to investigate the role of Na_2_SO_3_ in hemorrhagic shock resuscitation and found that injection of Na_2_SO_3_ within 24 h before resuscitation could increase the expression of glucocorticoid receptor (GCR) in tissue and abate the damage of lung mechanics and gas exchange. Swines with atherosclerosis were selected because CSE downregulation in this disease state is more conducive to reflecting the therapeutic effect of Na_2_SO_3_ [111,112]. Similarly, Na_2_SO_3_ treatment of CSE-deficient mice with ALI caused by blunt chest trauma and hemorrhage increased the expression of GCR and iNOS in the lung tissue [79]. These studies suggest that Na_2_SO_3_ may protect the lungs from ALI induced by blood loss by activating a mechanism that increases tissue GCR expression. This also indicates that Na_2_SO_3_ may be an effective treatment option for individuals with reduced CSE activity and/or insufficient endogenous H_2_S levels.

**Figure 5:**
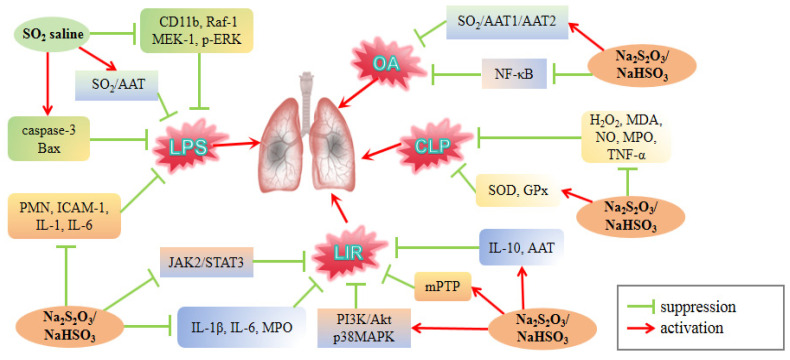
Regulation of ALI by SO_2_ donors. AAT: aspartate aminotransferase; LIR: limb ischemia-reperfusion; MEK-1: mitogen-activated protein kinase kinase 1; ERK: extracellular regulated protein kinases; JAK2: Janus kinase 2; STAT3: signal transducer and activator of transcription; mPTP: mitochondrial permeability transition pore. Two SO_2_ donors, SO_2_ saline and Na_2_S_2_O_3_/NaHSO_3_, improved ALI induced by LPS, OA and CLP, respectively, by inhibiting inflammation and anti-oxidation. Na_2_S_2_O_3_/NaHSO_3_ improved LIR-induced ALI by inhibiting the JAK2/STAT3 pathway and activating the PI3K/Akt and p38 MAPK pathways.

During LPS-induced lung injury in rats, the concentration of SO_2_ in the lung tissue and peripheral blood decreased significantly, and pre-administration of SO_2_ donors could prevent this reduction [82,83]. Zhai et al. [83] found that pretreatment with SO_2_ saline can significantly mitigate LPS-induced ALI in rats, lower the levels of IL-1β, IL-6, ICAM-1, and CD11b, and enhance the level of IL-10. However, the use of the AAT inhibitor L-aspartic acid-β-hydroxamic acid (HDX) can aggravate LPS-induced ALI (Table 1). These findings indicate that the reduction in the SO_2_/AAT pathway could be linked to the mechanism of LPS-induced lung damage. In addition, SO_2_ saline can inhibit the protein content of Raf-1, MEK-1, and phosphorylated extracellular signal-regulating protein kinase (p-ERK) in lung tissue [83], increase caspase-3 and Bax levels, reduce Bcl-2 protein synthesis, and promote PMN cell apoptosis in vivo and in vitro [82]. Given that the Raf/MEK/ERK signaling cascade influences cell proliferation, differentiation, development, and apoptosis [113,114], SO_2_ may protect against LPS-induced ALI by regulating cell apoptosis. Pretreatment with Na_2_SO_3_/NaHSO_3_ improved LPS-induced lung injury by reducing the number of PMN cells in BALF, decreasing ICAM-1 content in lung tissue, lowering IL-1β and IL-6 levels in serum, and increasing IL-10 concentrations in serum [81] (Figure 5). The above studies suggest that suppression of the SO_2_/AAT pathway may contribute to the development of LPS-induced lung damage, whereas exogenous SO_2_ supplementation can inhibit the inflammatory response in lung tissue and ameliorate ALI in rats.

The downregulation of endogenous AAT1/SO_2_ or AAT2/SO_2_ pathways in rats with OA-induced ALI is more noticeable, accompanied by increased ROS generation and decreased antioxidant capacity, which eventually leads to severe lung damage [76,84]. Treatment with Na_2_SO_3_/NaHSO_3_ decreased the generation of OH^−^ and O^2−^ within alveolar epithelial cells while significantly increasing the levels of SOD, GPx, and GSH. The application of HDX promoted the production of free radicals, further confirming that SO_2_ may protect against OA-induced ALI by suppressing oxidative stress [76]. Furthermore, Chen et al. [84] found that *AAT1* gene knockdown triggered NF-κB activation and an inflammatory response in human alveolar epithelial A549 cells in rats with OA-induced ALI, and this effect was abolished by NF-κB inhibitors. Notably, either SO_2_ donor (Na_2_SO_3_/NaHSO_3_) or AAT1 overexpression in vivo can protect lung tissue from OA-induced ALI by enhancing S-sulfenylation of NF-κB p65 at the cysteine 38 site and preventing NF-κB p65 activation [84].

Alveolar macrophages are the primary immune cells in alveoli, particularly during the inflammatory phase associated with ALI [115]. Zhao et al. [85] found that administering Na_2_SO_3_/NaHSO_3_ to rats with LIR-induced lung damage effectively decreased caspase-3 protein levels in alveolar macrophages while increasing Bcl-2 protein levels, ultimately preventing alveolar macrophage apoptosis. SO_2_ also inhibited the opening of the mPTP, increased mitochondrial membrane potential, reduced mitochondrial membrane permeability, and attenuated mitochondrial swelling. Mitochondrial dysfunction alters macrophage metabolism. Consequently, exogenous SO_2_ may improve alveolar macrophage apoptosis and minimize ALI damage via the mitochondrial pathway [116]. Given the overlap of H_2_S and SO_2_ synthetic pathways and similar biological effects, H_2_S may also act on alveolar macrophages. Studies have shown that blocking iNOS in macrophages can facilitate their repolarization from an inflammatory (M1) to an anti-inflammatory (M2) state by restoring mitochondrial oxidative phosphorylation, thereby contributing to disease management [115,116]. H_2_S decreases iNOS levels by inhibiting NF-κB p65 activation, leading to improved lung function [60,64]. Additionally, research has shown that H_2_S promotes M2 macrophage polarization by reducing iNOS levels in M1 macrophages or by increasing mitochondrial biogenesis and fatty acid oxidation [86,87]. Although all these investigations used bone marrow-derived macrophages, it is possible that H_2_S improves ALI by promoting alveolar macrophages polarization. Furthermore, studies have shown that natural compounds, such as resveratrol and tanshinone IIA, could potentially benefit ALI management [88]. At the same time, studies have reported that they are partially dependent on the effects of H_2_S in improving penile erectile dysfunction [117,118]. Consequently, H_2_S may be implicated in the treatment of ALI, a hypothesis that warrants further experimental investigation.

In addition, prophylactic and therapeutic administration of Na_2_SO_3_/NaHSO_3_ enhanced AAT activity in the lungs, decreased IL-1β and IL-6 levels, increased IL-10 levels, and decreased MPO activity [119,120]. Blocking SO_2_ formation with HDX exacerbates LIR-induced pulmonary inflammation [119]. Exogenous administration of SO_2_ donors has been shown to attenuate inflammation and lung damage caused by LIR by decreasing the release of inflammatory cytokines. Based on some evidence, Zhao et al. [120] used specific inhibitors of the PI3K/Akt, p38MAPK, and Janus kinase 2 (JAK2)/signal transducer and activator of transcription (STAT3) pathways, LY294002, SB03580 and Stattic, respectively, to verify the putative mechanism behind SO_2_’s protective effect on LIR-induced ALI. Following I/R, they found an elevation in the protein levels of p-STAT3, p-Akt, and p-p38 in rat lung tissues. After adding Na_2_SO_3_/NaHSO_3_, the levels of p-Akt and p-p38 proteins increased, while p-STAT3 levels decreased. Compared to the I/R + SO_2_ group, inhibition of the JAK2/STAT3 signaling pathway increased the protective effect of SO_2_, whereas blocking the PI3K/Akt and p38 MAPK signaling pathways reduced the protective effect. It should be noted that after Stattic administration, p-Akt levels increased but p-p38 levels remained unaltered. These findings indicate that the JAK2/STAT3, PI3K/Akt, and p38 MAPK pathways may contribute to SO_2_-induced ALI, with the JAK2/STAT3 pathway potentially influencing the PI3K/Akt pathway. In a rat model of CLP-induced ALI, Na_2_SO_3_/NaHSO_3_ intervention reduced the levels of H_2_O_2_, MDA, NO, MPO activity, and TNF-α in lung tissue, while increasing the levels of GPx and SOD activity [121]. SO_2_ has been demonstrated to improve the survival rate of patients with sepsis by mitigating sepsis-induced lung damage, and its mechanism involves the inhibition of oxidation triggered by lung injury and enhancement of antioxidant activity.

In conclusion, the downregulation of the AAT/SO_2_ pathway may play a role in the mechanism of LPS-induced lung injury. SO_2_ facilitates PMN apoptosis by suppressing the Raf/MEK/ERK pathway, leading to improved LPS-induced ALI. Both SO_2_ and AAT1 contribute to enhanced S-sulfenylation of NF-κB p65 at cys38, inhibiting p65 activation and thereby safeguarding lung tissue from OA-induced ALI. SO_2_ has been shown to mitigate mitochondrial swelling in LIR-induced ALI by inhibiting mPTP opening. Additionally, SO_2_ has been found to reduce LIR-induced inflammation and lung injury by inhibiting the JK2/STT3 pathway and activating the PI3K/AKT and P38 pathways. However, compared to H_2_S, there is a lack of comprehensive research data on the effects of SO_2_. Furthermore, the limited number of animal models utilized and the unclear specific mechanisms of action further contribute to this gap in knowledge. Given the similarities in metabolic pathways and biological effects between SO_2_ and H_2_S, it is plausible that SO_2_ may also have potential therapeutic benefits in ARDS induced by various factors such as smoke inhalation, hyperoxia, mechanical ventilation, PQ exposure, and resuscitation from hemorrhagic shock. In general, from the existing results, the effect of SO_2_ on ALI/ARDS is positive, but more in-depth exploration is lacking.

## 5. Conclusions and Perspective

This article provides an in-depth analysis of studies on sulfur-containing gas signaling molecules, such as H_2_S and SO_2_, and their potential role in mitigating histopathological lung damage in animal models of ALI induced by various factors. Previous studies have indicated that pulmonary changes and response to treatment of ARDS may vary depending on the subtype of ALI (direct vs. indirect factors), suggesting that personalized treatment approaches could improve treatment efficacy and patient survival rates. The anti-inflammatory, antioxidant, and anti-apoptotic properties of sulfur-containing gas signaling molecules contribute to their therapeutic efficacy in addressing multiple facets of ALI/ARDS pathogenesis, rendering them effective in both subtypes of the condition. During the acute exudative phase, diffuse alveolar damage in the lungs leads to the migration and activation of central granulocytes, resulting in the production of proinflammatory cytokines such as IL-1β, IL-6, IL-8, and TNF-α. H_2_S inhibits this process by suppressing the TLR4/MyD88/NF-κB pathway, while SO_2_ inhibits NF-κB p65 activation by S-sulfenylating it at Cys38, thus mitigating lung inflammation. Additionally, SO_2_ can suppress the JAK2/STAT3 pathway and activate the PI3K/Akt and p38 MAPK pathways to reduce levels of proinflammatory factors. Following type II alveolar cell injury, there is a reduction in the production of surfactant proteins SP-A and SP-B, resulting in decreased lung compliance and atelectasis. Treatment with H_2_S has been shown to elevate the levels of SP-A and SP-B in the bronchoalveolar region. The impairment of epithelial ion channel function hinders the reabsorption of edematous fluid from the alveoli to the stroma, while H_2_S treatment can enhance the expression of aquaporin proteins AQP1 and AQP5, thereby promoting the clearance of edematous fluid. Alveolar vascular injury can disrupt the normal vasomotor function, leading to the formation of microthrombi. Plasma levels of Ang2, P-selectin, and ICAM-1 are elevated in response to vascular endothelial cell (VEC) damage, with treatment using H_2_S and SO_2_ shown to decrease these levels. H_2_S inhibits the activation of p38 and NF-κB pathways, leading to reduced expression of ICAM-1 on the endothelial surface. Additionally, SO_2_ has been found to decrease ICAM-1 expression in lung tissue, although the precise mechanism remains unclear. The repair process from the exudative phase to the proliferative phase occurs gradually following VEC damage. However, progression to the fibrotic stage, known as late ARDS, may result in diffuse fibrotic changes in lung tissue. Endothelial cell proliferation contributes to elevated VEGF levels in plasma, with H_2_S demonstrating the ability to suppress VEGF release and VEGFR2 expression. Furthermore, oxidative stress, apoptosis, and autophagy are significant factors in the overall pathogenesis. Both H_2_S and SO_2_ have been shown to mitigate ROS production in lung tissue and enhance antioxidant enzyme expression, including SOD and GPx, thereby mitigating oxidative stress-induced damage. H_2_S also facilitates Nrf2 activation and upregulates enzyme expression, such as NQO1 and GST-A4. H_2_S plays a role in reducing apoptosis of alveolar cells through the activation of the PI3K/Akt pathway, while SO_2_ promotes apoptosis of PMN by inhibiting the Raf/MEK/ERK signaling pathway. Additionally, H_2_S inhibits autophagy and mitigates ALI by activating the PI3K/Akt/mTOR pathway. These results suggest that sulfur-containing gas signaling molecules show promise for the treatment of ALI.

Animal experiments outlined in this paper support the beneficial effects of sulfur-containing gas signaling molecules on ALI induced by LPS, OA, I/R, and sepsis. In contrast to the ALI model induced by gas inhalation, alternative models exhibit reduced relevance to the clinical setting. Nevertheless, these models possess common pathological features akin to those observed in clinical cases of ARDS. For instance, the administration of LPS can disrupt pulmonary epithelial and endothelial barriers, provoke the release and buildup of pro-inflammatory mediators, and prompt the infiltration of inflammatory cells, aligning with the pathogenesis of ALI/ARDS. LPS-induced ALI is frequently employed to replicate ARDS resulting from sepsis. Another example is OA, which is utilized to mimic ARDS induced by lipid embolism. Approximately 85% of intravenously administered OA is retained in the lungs and triggers the production of major inflammatory mediators seen in clinical ARDS, such as IL1, IL6, IL8, TNF-α and other major lung histological changes characteristic of ARDS. However, it is important to note that animal models may not fully replicate human diseases, but they are valuable for investigating the pathophysiology of ARDS. The use of larger animals in research studies necessitates higher drug doses to achieve similar effects as in smaller animals, which poses a risk of toxicity. This presents a challenge in translating research findings from animal models to larger animals and eventually to humans. However, the use of H_2_S treatment has shown promising outcomes in ALI models conducted in larger animal species, such as sheep and pigs. Consequently, conducting tests on novel drug candidates or alternative treatments using these models may lead to more dependable results, facilitating a smoother transition to clinical applications.

In summary, the use of sulfur-containing gas signaling molecules, such as H_2_S and SO_2_, has shown promise in ameliorating ALI stemming from various causes, and the development of pharmaceutical interventions targeting these molecules could provide a novel and advantageous approach for the clinical management and prevention of ARDS.

## Figures and Tables

**Table 1:** Regulatory effects of H_2_S and SO_2_ on different ALI models.

ALI Models	Donors	Mechanisms	Effects	Reference
LPS-induced ALI	NaHS	Regulating the composition and secretion of PS	SP-A mRNA expression↑	[43]
	NaHS	-	IL-1β, MDA and mitochondrial swelling↓;	[44]
			ATP enzyme, SOD and GPx activity↑	[45]
	GYY4137	Inhibiting TLR4/NF-κB/NLRP3 pathway	IL-1β, IL-6 and TNF-α↓	[46]
	GYY4137	Inhibiting Hoxb8 neutrophil pro-inflammatory signaling and oxidative burst	MIP-2, CXCR2 and IL-1β↓	[47]
	H_2_S gas	Inhibiting Nox2 and p38 MAPK pathway	HSP70, p-p38 MAPK, Nox2, IL-1β and ROS↓	[48]
	GYY4137	-	MDA, H_2_O_2_, 3-NT, iNOS, NO, IL-6, IL-8 and MPO↓; GSH/GSSG, T-AOC, catalase and SOD↑	[49]
	Allicin	Promoting PI3K/Akt pathway	PI3K, p-Akt and Bcl-2↑; caspase-3/-9 activity↓	[50]
	NaHS	Inhibiting p38 MAPK pathway	PMN, MPO, MDA, ICAM-1 and p-p38 MAPK↓; SOD activity↑	[51]
	Allicin	Inhibiting TLR4/MyD88/NF-κB pathway	TNF-α, IL-6 and IL-1β↓	[52]
	H_2_S gas	Inhibiting neutrophil migration and pro-inflammatory cytokine release	IL-1β, MIP-2 and MPO↓	[53]
	GYY4137	Promoting PI3K/Akt/mTOR pathway	TNF-α, IL-1β and protein content in BALF↓	[54]
	DATS	Down-regulating NF-κB expression	NF-κB activity and TNF-α mRNA expression ↓	[55]
	NaHS	-	PMN, MDA and MPO↓	[56]
	NaHS	-	iNOS and NO↓	[41,42]
	NaHS	Inhibiting NF-κB pathway	PMN, ICAM-1 and NF-κB↓	[57,58]
	GYY4137	Up-regulating HO-1 expression	iNOS and COX-2↓	[59]
Inhalation-induced ALI	H_2_S gas	Inhibiting the activation of NF-κB p65	MDA, NO, NF-κB p65, iNOS and iNOS mRNA expression↓	[60]
	Na_2_S	-	IL-6 and IL-8↓; IL-10↑	[61]
	Na_2_S	-	iNOS, MPO and PARP-1↓	[62]
Hyperbaric hyperoxia-induced ALI	NaHS	-	TUNEL positive cells, protein in BALF and IL-13↓	[63]
	NaHS	-	Ang2, MDA, ROS, IL-1β, MCP-1, MIP-2, NF-κB, iNOS, Nox, VEGF and VEGFR2↓	[64]
	NaHS	-	Ang2, MDA, ROS, IL-1β, MCP-1 and MIP-2	[65]
Ventilator-induced ALI	Na2S	-	CXCL-2, CD11b and IL-6↓; NQO1, GPx2 and GST-A4↑	[66]
OA-induced ALI	NaHS	-	Fas protein expression↓	[67]
	NaHS	Up-regulating endoplasmic reticulum stress proteins	GRP78 and eIF2α↑	[68]
	NaHS	-	IL-6 and IL-8↓; IL-10↑	[69]
	NaHS	-	PMN, NF-κB and ICAM-1↓	[70]
	NaHS	-	MDA↑; SOD and GSH↓	[71]
RIR-induced ALI	NaHS	Promoting NRF2 activation-mediated inhibition of NLRP3 pathway	HO-1, NQO1 and Trx↑; NLRP3, caspase-1 and IL-1β↓	[72]
IAC-induced ALI	GYY4137	Promoting p-Akt and the activation of GSK-3β and S6K	Ang2, MPO, TNF-α, IL-6 and IL-1β↓	[73]
LIR-induced ALI	NaHS	Inhibiting TLR4/MyD88/NF-κB pathway and AQP1/AQP5	TLR4, MyD88, p-NF-κB, AQP1 and AQP5↓	[74]
PQ-induced ALI	DAS	-	NO and iNOS mRNA expression↓	[75]
	DAS	-	NF-κB p65 and TNF-α mRNA↓	[76]
CLP-induced ALI	GYY4137	Inhibiting PDGFRβ/Akt/NF-κB/NLRP3 pathway	p-PDGFRβ, p-NF-κB, ASC, NLRP3, caspase-1 and p-Akt↓	[77]
CLP-/LPS-induced ALI	GYY4137	Promoting PI3K/Akt/mTOR pathway	p-mTOR and Beclin1↓; LC3II/LC3I, GPx4 and SLC7A11↑	[78]
Caerulein-induced acute pancreatitis-related ALI	NaHS	-	MPO, CCL2, CXCL1, ICAM-1, VCAM-1↓	[25]
	ACS15	-	MPO activity↓	[24]
Blunt chest trauma and bleeding-induced ALI	Na_2_S_2_O_3_	-	GCR and iNOS↑	[79]
Hemorrhagic shock-induced ALI	Na_2_S_2_O_3_	-	GCR expression↑	[80]
LPS-induced ALI	Na_2_SO_3_/ NaHSO_3_	-	PMN, ICAM-1, IL-1, IL-6↓; IL-10↑	[81]
	SO_2_ saline	-	caspase-3 and Bax↑; Bcl-2↓	[82]
	SO_2_ saline	Up-regulating SO_2_/AAT pathway	IL-1β, IL-6, ICAM-1, CD11b, Raf-1, MEK-1, p-ERK↓; IL-10↑	[83]
OA-induced ALI	Na_2_SO_3_/ NaHSO_3_	Up-regulating SO_2_/AAT1/AAT2 pathway and enhancing sulphenylation of NF-κB p65 at cysteine 38	SOD, GPx, GSH↑; OH^−^ and O^2−^↓	[76,84]
LIR-induced ALI	Na_2_SO_3_/ NaHSO_3_	Inhibiting opening of mPTP	AM apoptosis and Caspase-3 expression↓; Bcl-2↑	[85]
	Na_2_SO_3_/ NaHSO_3_	-	IL-1β, IL-6 and MPO↓; IL-10 and AAT activity↑	[86]
	Na_2_SO_3_/ NaHSO_3_	Inhibiting JAK2/STAT3 and promoting PI3K/Akt and p38MAPK pathway	p-Akt, p-p38 ↑; p-STAT3 ↓	[87]
CLP-induced ALI in SD rats	Na_2_SO_3_/ NaHSO_3_	-	H_2_O_2_, MDA, NO, MPO and TNF-α↓; SOD and GPx↑	[88]

## Abbreviations

3-NT	3-nitrotyrosine
ASC	Apoptosis-associated speck-like protein containing a CARD
CCL2	Chemokine (C-C motif) ligand 2
COX-2	Cyclooxygenase-2
CXCL-2	Chemokine (C-X-C motif) ligand 2
CXCR2	C-X-C motif-chemokine receptor 2
eIF2α	Eukaryotic translation initiation factor-2
GRP 78	Glucose-regulated protein 78
GSK-3β	Glycogen synthase kinase 3β
GSSG	Oxidative glutathione
GST-A4	Glutathione-s-transferase a4
H_2_O_2_	Hydrogen peroxide
HSP 70	Heat shock protein 70
IFN-γ	Interferon-γ
Keap1	Kelch-like ECH-associated protein 1
MCP-1	Macrophage chemoattractant protein-1
MEK	Mitogen-activated protein kinase
mPTP	Mitochondrial permeability transition pore
NQO1	NAD(P)H: quinone oxidoreductase
PARP-1	Poly (ADP-ribose) polymerase-1
PDGFRβ	Platelet-derived growth factor Rβ-chain
S6K	Ribosomal protein s6 kinase
SLC7A11	Solute carrier family 7 member 11
SnPP	Tin protoporphyrin
T-AOC	Total antioxidant capacity
Trx	Thioredoxin
VCAM-1	Vascular cell adhesion molecule-1
VEGF	Vascular endothelial growth factor
VEGFR2	Vascular endothelial growth factor receptor 2
γ-GCS	Gamma glutamylcysteine synthetase
